# Blood pressure variability and parenchymal hematoma after endovascular thrombectomy: effect modification by recanalization status and cerebral small vessel disease burden

**DOI:** 10.3389/fneur.2026.1927081

**Published:** 2026-09-10

**Authors:** Meng-Ni Wu, Yu-Peng Liu, Wei-Hao Lin, I-Hsiao Yang, Sui-Sum Kung, Chung-Yao Hsu, Hsiu-Fen Lin

**Affiliations:** 1 Graduate Institute of Clinical Medicine, College of Medicine, Kaohsiung Medical University, Kaohsiung, Taiwan; 2 Department of Neurology, Kaohsiung Medical University Hospital, Kaohsiung, Taiwan; 3 Department of Neurology, College of Medicine, Kaohsiung Medical University, Kaohsiung, Taiwan; 4 Department of Medical Imaging, Kaohsiung Medical University Hospital, Kaohsiung, Taiwan; 5 Department of Neurosurgery, Kaohsiung Medical University Hospital, Kaohsiung, Taiwan

**Keywords:** blood pressure variability, cerebral small vessel disease, endovascular thrombectomy, parenchymal hematoma, recanalization

## Abstract

**Background:**

We investigated the association between post-thrombectomy blood pressure variability (BPV) and parenchymal hematoma (PH), and whether this relationship is modified by persistent vessel patency and cerebral small vessel disease (CSVD) burden.

**Methods:**

We analyzed 302 patients with acute ischemic stroke who underwent endovascular thrombectomy. PH was defined as confluent hemorrhage with a mass effect. BPV was assessed using 2-h blood pressure measurements during the first 24 h. Parameters included the maximum, minimum, mean, range, standard deviation, coefficient of variation, and successive variation of systolic, diastolic, and pulse pressure (PP). CSVD burden was quantified using the total CSVD score and dichotomized into none-to-mild (score 0–1) and moderate-to-severe (score 2–4). Multivariable logistic regression models were used to evaluate associations and interaction effects.

**Results:**

PH occurred in 78 patients (25.8%). Among BPV parameters, pulse pressure successive variation (PP_SV_) showed favorable model performance and was selected for interaction analyses. Significant interactions were observed between PP_SV_ and persistent vessel patency (*P_interaction_* = 0.003) and between PP_SV_ and CSVD burden (*P_interaction_* = 0.007). In stratified analyses, higher PP_SV_ was associated with PH in patients with sustained major vessel patency (OR, 2.67 [95% CI, 1.79–3.97]; *p* < 0.001) and in those with none-to-mild CSVD (OR, 2.61 [95% CI, 1.74–3.94]; *p* < 0.001) but not in those with persistent occlusion or moderate-to-severe CSVD.

**Conclusion:**

Increased post-thrombectomy BPV, particularly PP variability, was independently associated with PH, with effects modified by persistent vessel patency and CSVD burden. These findings suggest individualized blood pressure management based on macrovascular persistent vessel patency and underlying microvascular integrity.

## 1 Introduction

Endovascular thrombectomy (EVT) has substantially improved clinical outcomes in patients with acute ischemic stroke caused by large-vessel occlusion. However, parenchymal hematoma (PH) remains one of the most devastating complications and is strongly associated with poor functional outcomes and increased mortality (1). Identifying modifiable risk factors for PH is therefore critical for post-thrombectomy management.

Physiologically, an intact blood–brain barrier (BBB) and balanced hydrostatic and osmotic pressures prevent the extravasation of blood constituents (2, 3). When ischemia compromises BBB integrity, the unrestricted movement of solutes diminishes the osmotic gradient. Consequently, blood pressure, as the primary determinant of hydrostatic pressure, may contribute to blood extravasation into infarcted tissue, ultimately leading to hemorrhagic transformation and PH (2–4).

However, post-thrombectomy BP management is complex, as intensive BP lowering does not necessarily improve outcomes. Accordingly, the updated European Stroke Organisation (ESO) and American Heart Association/American Stroke Association (AHA/ASA) guidelines recommend against intensive SBP lowering below 140 mmHg during the early post-thrombectomy period (5, 6). Beyond absolute BP levels, increased blood pressure variability (BPV) may exert repetitive hemodynamic stress on the vulnerable microvasculature, potentially aggravating BBB disruption and contributing to PH (4, 7–9). Nevertheless, the association between post-thrombectomy BPV and PH remains inconsistent across studies, potentially due to heterogeneous patient characteristics that influence vascular vulnerability (9–14).

Reperfusion after ischemia can exacerbate endothelial injury and inflammatory responses, compromising BBB integrity and increasing the risk of hemorrhagic transformation (2, 15). In addition, cerebral small vessel disease (CSVD), which is characterized by chronic endothelial dysfunction and BBB impairment, has been associated with hemorrhagic complications after intravenous thrombolysis (16–18). A higher global CSVD burden has also been reported to correlate with increased BBB leakage (19). Consequently, varying CSVD burdens may reflect disparate levels of vulnerability to blood pressure-driven extravasation after ischemic stroke.

We hypothesized that the relationship between post-thrombectomy BPV and PH is contingent upon both macrovascular persistent vessel patency and baseline microvascular integrity reflected by the CSVD burden. Therefore, this study aimed to investigate the association between BPV within 24 h following EVT and PH and to determine whether this relationship is modified by persistent vessel patency and CSVD burden.

## 2 Materials and methods

In this retrospective study, we reviewed patients with acute ischemic stroke who underwent EVT and who were admitted to the neurointensive care unit at Kaohsiung Medical University Hospital between January 2018 and July 2025. The study was approved by the Institutional Review Board of Kaohsiung Medical University Hospital (KMUHIRB-E(I)-20260069). The study was conducted in accordance with the ethical principles of the Declaration of Helsinki. The requirement for written informed consent was waived by the Institutional Review Board because of the retrospective nature of the study.

### 2.1 Participants

Patients were included if they met the following criteria: (1) age ≥20 years; (2) acute ischemic stroke with large vessel occlusion (LVO) involving the anterior circulation, including the internal carotid artery, middle cerebral artery or anterior cerebral artery; and (3) complete magnetic resonance imaging (MRI) data, including magnetic resonance angiography (MRA), diffusion-weighted imaging, T2-weighted imaging (T2WI), T2-fluid-attenuated inversion recovery (T2-FLAIR), T1-weighted imaging (T1WI), and susceptibility-weighted imaging (SWI). Patients who were unable to undergo complete MRI because of clinical instability, early neurological deterioration requiring urgent management, contraindications to MRI, poor general condition, or death before the scheduled MRI examination were not enrolled in this study.

The exclusion criteria included (1) contrast extravasation on digital subtraction angiography; (2) preexisting vasculopathies (e.g., cerebral amyloid angiopathy, tumor, or vasculitis); (3) known bleeding diathesis; and (4) metabolic or systemic derangements potentially masking neurological assessment, such as profound shock, severe hypernatremia (>155 mmol/L), hyponatremia (<125 mmol/L), or hypoglycemia (<60 mg/dL) on admission.

All patients underwent assessments using the National Institutes of Health Stroke Scale (NIHSS) and brain computed tomography (CT) with CT angiography (CTA) before EVT. After admission, evaluations for cardiovascular risk factors, 24-h ambulatory electrocardiogram monitoring, and brain MRI were performed. Follow-up brain CT scans were obtained if neurological deterioration was observed. The demographic characteristics, medical history, use of intravenous thrombolysis, recanalization status estimated by the modified thrombolysis in cerebral infarction (mTICI) score, onset-to-puncture time (OTP), and collateral circulation status were retrieved from medical records. Collateral circulation was evaluated via CTA and graded according to the Tan collateral score, with good collateral circulation defined as filling > 50% of the occluded territory (20). The extent of infarction was assessed on brain MRI using the Alberta Stroke Program Early CT Score (ASPECTS) (21). To avoid confounding from re-occlusion after successful recanalization, MRA performed 2–4 days after stroke onset was used to assess status of vessel patency. The modified arterial occlusive lesion (AOL) classification grade 2–3 was defined as sustained major vessel patency (22).

### 2.2 Definition of parenchymal hematoma

PH was assessed according to a predefined imaging protocol. Patients with neurological deterioration underwent immediate non-contrast CT followed by the scheduled MRI, whereas patients without neurological deterioration underwent the scheduled MRI alone. Hyperdense lesions immediately after EVT were not considered PH unless hemorrhage was confirmed on follow-up imaging. The readers were blinded to clinical information. PH was defined as hemorrhage with a mass effect identified on MRI or follow-up CT. It was subdivided into PH-1 (hemorrhage involving ≤ 30% of the infarcted area with mild mass effect) and PH-2 (hemorrhage involving > 30% of the infarcted area or extending beyond it with significant mass effect) (23).

### 2.3 Blood pressure variability parameters

Systolic blood pressure (SBP) and diastolic blood pressure (DBP) were automatically measured every 2 h for the first 24 h after EVT completion upon admission to the neurocritical care unit using a noninvasive automated arm-cuff blood pressure monitoring device (IntelliVue MX700; Philips), yielding 12 BP measurements per patient. Pulse pressure (PP) was calculated as the difference between SBP and DBP. Mean blood pressure (MBP) was calculated as (SBP + 2 × DBP)/3. The BPV parameters included the maximum (max), minimum (min), and mean values derived from repeated measurements over the first 24 h. Additional BPV parameters, including range (maximum–minimum), standard deviation (SD), coefficient of variation (CV), and successive variation (SV), were calculated. All BPV parameters were calculated separately for SBP, DBP, PP, and MBP. Because BP measurements were automatically recorded by the bedside monitoring system at predefined intervals, no BP data were missing. For patients who underwent imaging examinations or surgical intervention for neurological deterioration during the monitoring period, BP measurements obtained from portable monitoring devices and documented in anesthesia or operative records were used to ensure complete BP recording. Post-thrombectomy BP management followed institutional practice based on contemporary stroke guidelines, with systolic BP generally maintained below 180 mmHg. For patients with successful reperfusion, lower BP targets were considered at the treating physician’s discretion. Blood pressure management, including antihypertensive treatment, followed routine clinical practice rather than a standardized study protocol. EVT was performed under general anesthesia in all patients, and subsequent anesthesia management in the neurocritical care unit followed routine clinical practice. The formulas used to derive these BPV parameters are provided below (4, 8, 9).

$$\mathrm{SD}=\sqrt{\;\frac{1}{n-1}\sum_{i=1}^{n}{\left(\mathrm{BPi}-\mathrm{BP}\;\text{mean}\right)}^{2}\;}$$

$$\mathrm{CV}=\frac{\mathrm{SD}}{\text{mean}\;\mathrm{BP}}\times 100\%$$

$$\mathrm{SV}=\sqrt{\frac{1}{n-1}\sum_{i=1}^{n-1}{\left(\mathrm{BPi}+1-\mathrm{BPi}\right)}^{2}\;}$$

### 2.4 Cerebral small vessel disease (CSVD)

All MRI examinations were performed using a 3.0-T scanner (GE Healthcare). A board-certified neurologist (W. H. L.), blinded to clinical and blood pressure data, reviewed all sequences. CSVD markers, including lacunes, white matter hyperintensities (WMH), enlarged perivascular spaces (EPVS), and cerebral microbleeds (CMB) according to the STandards for Reporting Vascular Changes on Neuroimaging (STRIVE) criteria (24). To minimize potential interference from acute ischemic lesions, cerebral edema, hemorrhagic transformation, and post-procedural imaging changes, CSVD markers were assessed in the contralateral hemisphere whenever applicable, thereby providing a more reliable estimate of the pre-existing CSVD burden.

According to the scoring system developed by Staals et al., the total CSVD score was used to quantify the cumulative CSVD burden, which ranged from 0 to 4 (25). One point was assigned for the presence of each of the following imaging markers: (1) ≥ 1 lacune; (2) ≥ 1 CMB; (3) moderate-to-severe EPVS, defined as >10 punctates (< 3 mm) or linear CSF-signal structures in the basal ganglia; and (4) severe WMH, defined as a Fazekas score of 2–3 in deep white matter (Deep-WMH) or 3 in the periventricular region (PV-WMH) (25–27). Based on prior studies, the total CSVD score was dichotomized into none-to-mild CSVD (score 0–1) and moderate-to-severe CSVD (score ≥ 2) groups for analytical purposes (28–30).

To assess intra-rater reliability, a random subset of 70 brain MRI scans was re-evaluated by the same neurologist after a 4-week interval, blinded to all prior ratings and clinical data. For the dichotomous variables, Cohen’s kappa coefficients were 0.765 for lacunes, and 0.928 for CMB. For ordinal and continuous parameters, the intraclass correlation coefficients using a two-way mixed-effects model were as follows: 0.872 (95% CI, 0.802–0.918) for PV-WMH, 0.910 (95% CI, 0.859–0.943) for Deep-WMH, and 0.951 (95% CI, 0.921–0.969) for EPVS.

### 2.5 Statistical analysis

Statistical analyses were performed using IBM SPSS Statistics for Windows, Version 22.0 (IBM Corp., Armonk, NY, USA). Continuous variables are presented as the mean ± SD or median (interquartile range [IQR]) on the basis of their distribution and were compared using Student’s *t* test or the Mann–Whitney *U* test, as appropriate. Categorical variables are reported as numbers (percentages) and were compared using the chi-square test. Univariable analyses were conducted to investigate the associations of clinical risk factors and individual BPV parameters with PH. Multivariable logistic regression models were constructed to identify independent associations between BPV parameters and PH, adjusting for variables with a *p* < 0.2 in the univariable analyses. To facilitate direct comparisons, all BPV parameters were standardized and analyzed per 1-SD increase. Interaction effects between BPV and persistent vessel patency or CSVD burden on PH were assessed by including interaction terms (BPV × persistent vessel patency and BPV × CSVD burden) in multivariable logistic regression models to calculate the *p* for interaction (*P_interaction_*). A two-sided *p* < 0.05 was considered to indicate statistically significant.

## 3 Results

### 3.1 Baseline demographic and clinical characteristics of the study population

Among the 437 patients with acute ischemic stroke who underwent EVT during the study period, 135 were excluded for the following reasons: non-anterior circulation occlusion (*n* = 68), incomplete MRI data (*n* = 61), contrast extravasation on digital subtraction angiography (*n* = 4), probable cerebral amyloid angiopathy (*n* = 1), and post-thrombectomy Takotsubo cardiomyopathy with profound shock (*n* = 1). Consequently, 302 patients were included in the final analysis.

The median age was 72 years (IQR, 63–80), and 170 patients (56.3%) were men. PH occurred in 78 patients (25.8%), including PH-1 (*n* = 44) and PH-2 (*n* = 34). Compared with patients in the non-PH group, patients in the PH group had a higher prevalence of sustained major vessel patency (80.8% vs. 62.9%; *p* = 0.004) and atrial fibrillation (76.9% vs. 52.7%; *p* < 0.001). Regarding stroke severity, the PH group had higher baseline NIHSS scores (18 [IQR, 14–22] vs. 16 [IQR, 12–20]; *p* = 0.017) and lower ASPECTS (5 [IQR, 2–7] vs. 7 [IQR, 3–8]; *p* < 0.001). Additionally, a moderate-to-severe CSVD burden was significantly higher in the PH group than in the non-PH group (53.8% vs. 25.4%; *p* < 0.001). The burden of individual CSVD markers, including lacunes (35.9% vs. 14.7%; *p* < 0.001), CMB (29.5% vs. 10.3%; *p* < 0.001), moderate-to-severe EPVS (76.9% vs. 58.9%; *p* = 0.004), and severe WMH (29.5% vs. 13.8%; *p* = 0.002), were greater in the PH group. Table 1 shows the baseline demographic and clinical characteristics.

**Table 1:** Baseline demographic and clinical characteristics of the study population.

Characteristics	Total (*n* = 302)	PH (*n* = 78)	Non-PH (*n* = 224)	*p* value
Age, years, median (IQR)	72.0 (63.0–80.0)	74.0 (65.0–80.0)	71.0 (63.0–79.5)	0.232
Sex (male), *n* (%)	170 (56.3)	41 (52.6)	129 (57.6)	0.441
Intravenous thrombolysis, *n* (%)	115 (38.1)	24 (30.8)	91 (40.6)	0.123
Onset-to-puncture time, minutes, median (IQR)	335.0 (254.0–446.0)	357.0 (244.0–460.0)	332.5 (254.3–445.8)	0.435
Good Collateral circulation, *n* (%)	158 (52.3)	35 (44.9)	123 (54.9)	0.126
Successful recanalization, *n* (%)	281 (93.0)	74 (94.9)	207 (92.4)	0.462
Sustained major vessel patency, *n* (%)	204 (67.5)	63 (80.8)	141 (62.9)	0.004*
ASPECTS, median (IQR)	7 (3–8)	5 (2–7)	7 (3–8)	<0.001*
NIHSS, score, median (IQR)	16 (12–21)	18 (14–22)	16 (12–20)	0.017*
Total CSVD score, median (IQR)	1 (0–2)	2 (1–3)	1 (0–2)	<0.001*
Lacunes, *n* (%)	61 (20.2)	28 (35.9)	33 (14.7)	<0.001*
CMB, *n* (%)	46 (15.2)	23 (29.5)	23 (10.3)	<0.001*
Moderate-to-Severe EPVS, *n* (%)	192 (63.6)	60 (76.9)	132 (58.9)	0.004*
Severe WMH, *n* (%)	54 (17.9)	23 (29.5)	31 (13.8)	0.002*
Moderate to Severe CSVD, *n* (%)	99 (32.8)	42 (53.8)	57 (25.4)	<0.001*
Diabetes mellitus, *n* (%)	83 (27.5)	22 (28.2)	61 (27.2)	0.868
Hypertension, *n* (%)	211 (69.9)	52 (66.7)	159 (71.0)	0.474
Dyslipidemia, *n* (%)	121 (40.1)	31 (39.7)	90 (40.2)	0.946
Atrial fibrillation, *n* (%)	178 (58.9)	60 (76.9)	118 (52.7)	<0.001*
Anti-platelet or anti-coagulant, *n* (%)	106 (35.1)	29 (37.2)	77 (34.4)	0.655
Current smoker, *n* (%)	43 (14.2)	11 (14.1)	32 (14.3)	0.968
White blood count, 10^3^/ul, median (IQR)	8.6 (6.8–10.6)	9.0 (7.0–11.0)	8.6 (6.8–10.4)	0.400
Platelet, 10^3^/ul, median (IQR)	188.5 (153.0–231.0)	181.0 (155.0–238.0)	190.0 (153.0–230.5)	0.796
eGFR (ml/min/1.73 m^2^), mean (±SD)	80.2 (31.1)	80.0 (30.7)	80.3 (31.3)	0.957
Proteinuria, *n* (%)	125 (41.4)	37 (47.4)	88 (39.3)	0.208

^*^p < 0.05.

PH, parenchymal hematoma; IQR, interquartile range; SD, standard deviation; ASPECTS, Alberta Stroke Program Early CT Score; NIHSS, the National Institutes of Health Stroke Scale; CSVD, cerebral small vessel disease; CMB, cerebral microbleed; EPVS, enlarged perivascular spaces; WMH, white matter hyper-intensities; eGFR, estimated glomerular filtration rate.

### 3.2 Comparison of blood pressure variability parameters between patients with and without parenchymal hematoma

Table 2 presents the associations between BPV parameters and PH. Patients with PH exhibited significantly higher DBP_CV_ (15.8% [IQR, 13.6–18.5] vs. 14.5% [11.5–18.2]; *p* = 0.030) and DBP_SV_ (12.2 [9.9–14.7] vs. 11.1 [9.1–13.9] mmHg; *p* = 0.015). Several PP variability indices were also significantly higher in the PH group, including the PP_max-min_ (44.5 [37.8–55.5] vs. 39.0 [30.3–54.0] mmHg; *p* = 0.003), PP_SD_ (11.2 [9.4–13.4] vs. 9.9 [8.0–13.0] mmHg; *p* = 0.012), PP_CV_ (20.5% [17.9–26.8] vs. 19.5% [14.9–25.1]; *p* = 0.015), and PP_SV_ (14.0 [11.3–16.6] vs. 10.5 [8.5–14.0] mmHg; *p* < 0.001). Additionally, MBP_SV_ was significantly higher in patients with PH than in those without PH (12.2 [10.2–14.8] vs. 11.4 [9.1–13.8] mmHg; *p* = 0.013). In contrast, while not reaching statistical significance, SBP_SV_ tended to be associated with PH (15.6 [13.5–19.2] vs. 15.3 [12.4–18.7] mmHg; *p* = 0.084).

**Table 2:** Comparison of blood pressure variability parameters between patients with and without parenchymal hematoma.

Blood pressure parameters	PH (*n* = 78)	Non-PH (*n* = 224)	*p* value
SBP			
SBP_mean_, mmHg, mean (±SD)	123.6 (11.8)	125.3 (13.0)	0.325
SBP_max_, mmHg, median (IQR)	159.0 (144.8–173.0)	158.0 (145.3–172.8)	0.926
SBP_min_, mmHg, mean (±SD)	97.8 (13.4)	99.4 (13.1)	0.365
SBP_max-min_, mmHg, median (IQR)	59.0 (46.8–76.3)	57.0 (47.0–72.8)	0.392
SBP_SD_, mmHg, median (IQR)	15.0 (12.2–17.5)	15.2 (11.7–18.0)	0.999
SBP_CV,_ %, median (IQR)	12.2 (10.4–14.3)	12.0 (9.5–14.6)	0.725
SBP_SV_, mmHg, median (IQR)	15.6 (13.5–19.2)	15.3 (12.4–18.7)	0.084
DBP			
DBP_mean_, mmHg, mean (±SD)	69.4 (11.4)	71.1 (10.5)	0.251
DBP_max_, mmHg, median (IQR)	97.5 (80.5–113.3)	97.0 (88.0–106.8)	0.831
DBP_min_, mmHg, mean (±SD)	52.3 (10.6)	54.4 (10.4)	0.132
DBP_max-min_, mmHg, median (IQR)	43.5 (33.0–57.0)	40.0 (33.0–52.8)	0.290
DBP_SD_, mmHg, median (IQR)	10.9 (8.5–13.4)	10.0 (8.4–12.5)	0.106
DBP_CV_, %, median (IQR)	15.8 (13.6–18.5)	14.5 (11.5–18.2)	0.030*
DBP_SV_, mmHg, median (IQR)	12.2 (9.9–14.7)	11.1 (9.1–13.9)	0.015*
PP			
PP_mean_, mmHg, median (IQR)	55.0 (44.5–64.1)	51.6 (45.2–62.7)	0.774
PP_max_, mmHg, median (IQR)	79.0 (65.0–89.0)	73.0 (64.0–89.8)	0.204
PP_min_, mmHg, median (IQR)	31.5 (22.5–42.0)	34.0 (24.3–42.0)	0.269
PP_max-min_, mmHg, median (IQR)	44.5 (37.8–55.5)	39.0 (30.3–54.0)	0.003*
PP_SD_, mmHg, median (IQR)	11.2 (9.4–13.4)	9.9 (8.0–13.0)	0.012*
PP_CV_, %, median (IQR)	20.5 (17.9–26.8)	19.5 (14.9–25.1)	0.015*
PP_SV_, mmHg, median (IQR)	14.0 (11.3–16.6)	10.5 (8.5–14.0)	<0.001*
MBP			
MBP_mean_, mmHg, mean (±SD)	88.6 (10.5)	88.5 (8.8)	0.935
MBP_max_, mmHg, median (IQR)	116.7 (103.3–129.8)	113.8 (105.8–123.5)	0.205
MBP_min_, mmHg, mean (±SD)	70.4 (10.8)	70.5 (9.6)	0.939
MBP_max-min_, mmHg, median (IQR)	44.8 (37.0–58.7)	43.0 (34.3–53.6)	0.148
MBP_SD_, mmHg, median (IQR)	11.2 (9.3–13.4)	10.7 (8.8–13.2)	0.159
MBP_CV,_ %, median (IQR)	12.6 (10.9–14.6)	12.1 (9.9–15.0)	0.162
MBP_SV_, mmHg, median (IQR)	12.2 (10.2–14.8)	11.4 (9.1–13.8)	0.013*

^*^p < 0.05.

PH, parenchymal hematoma; IQR, interquartile range; SBP, systolic blood pressure; DBP, diastolic blood pressure; PP, pulse pressure; MBP, mean blood pressure; max, maximum; min, minimum; max-min, the difference between maximum and minimum; SD, standard deviation; CV, coefficient of variation; SV, successive variation.

After adjustment for potential confounders identified in univariable analyses, several PP variability indices remained independently associated with PH (Table 3). These included PP_max-min_ (odds ratio [OR], 1.48; 95% CI, 1.09–2.00; *p* = 0.011), PP_SD_ (OR, 1.41; 95% CI, 1.04–1.90; *p* = 0.025), PP_CV_ (OR, 1.50; 95% CI, 1.11–2.01; *p* = 0.008), and PP_SV_ (OR, 1.86; 95% CI, 1.38–2.50; *p* < 0.001). Although not reaching statistical significance, SBP_SV_ (OR, 1.33; 95% CI, 0.99–1.78; *p* = 0.055) and DBP_SV_ (OR, 1.29; 95% CI, 0.97–1.71; *p* = 0.084) tended to be associated with PH after adjustment. The baseline clinical model yielded a C-statistic of 0.77 (95% CI, 0.71–0.84). Among all BPV parameters, PP_SV_ demonstrated the best overall model performance, with the highest C-statistic (0.81, 95% CI, 0.75–0.87), the lowest AIC (327.1), and a significant LRT (*p* < 0.001); therefore, it was selected for subsequent interaction analyses.

**Table 3:** Multivariable associations between blood pressure variability parameters (per 1-SD increase) and parenchymal hematoma after endovascular thrombectomy.

BPV parameters (per 1-SD increase)	Adjusted OR (95% CI)	*p* value	C-statistic (95% CI)	AIC	LRT *p* value
SBP parameters					
SBP_mean_	0.91 (0.68–1.22)	0.524	0.78 (0.71–0.84)	348.1	0.522
SBP_max_	1.01 (0.75–1.36)	0.955	0.77 (0.71–0.84)	349.0	0.955
SBP_min_	0.93 (0.70–1.25)	0.648	0.78 (0.71–0.84)	348.2	0.648
SBP_max-min_	1.06 (0.78–1.43)	0.705	0.77 (0.71–0.84)	348.5	0.706
SBP_SD_	1.02 (0.75–1.37)	0.924	0.77 (0.71–0.84)	349.0	0.924
SBP_CV_	1.05 (0.78–1.41)	0.747	0.77 (0.71–0.84)	348.9	0.748
SBP_SV_	1.33 (0.99–1.78)	0.055	0.78 (0.71–0.84)	344.8	0.056
DBP parameters					
DBP_mean_	0.97 (0.73–1.30)	0.848	0.77 (0.71–0.84)	347.7	0.848
DBP_max_	1.03 (0.78–1.36)	0.852	0.77 (0.71–0.84)	347.6	0.852
DBP_min_	1.02 (0.76–1.37)	0.909	0.77 (0.71–0.84)	345.4	0.909
DBP_max-min_	1.02 (0.77–1.36)	0.890	0.77 (0.71–0.84)	347.4	0.891
DBP_SD_	1.15 (0.86–1.53)	0.350	0.77 (0.71–0.84)	345.9	0.351
DBP_CV_	1.15 (0.86–1.54)	0.353	0.77 (0.71–0.84)	344.6	0.353
DBP_SV_	1.29 (0.97–1.71)	0.084	0.78 (0.72–0.84)	342.5	0.083
PP parameters					
PP_mean_	0.94 (0.70–1.25)	0.654	0.78 (0.71–0.84)	349.0	0.653
PP_max_	1.16 (0.86–1.56)	0.323	0.77 (0.71–0.84)	347.4	0.324
PP_min_	0.78 (0.59–1.04)	0.090	0.78 (0.72–0.85)	347.2	0.086
PP_max-min_	1.48 (1.09–2.00)	0.011*	0.79 (0.72–0.85)	342.9	0.011*
PP_SD_	1.41 (1.04–1.90)	0.025*	0.79 (0.73–0.85)	344.3	0.024*
PP_CV_	1.50 (1.11–2.01)	0.008*	0.80 (0.74–0.85)	343.3	0.007*
PP_SV_	1.86 (1.38–2.50)	<0.001*	0.81 (0.75–0.87)	327.1	<0.001*
MBP parameters					
MBP_mean_	1.18 (0.86–1.61)	0.298	0.78 (0.71–0.84)	349.0	0.209
MBP_max_	1.17 (0.87–1.57)	0.308	0.78 (0.71–0.84)	347.1	0.220
MBP_min_	1.26 (0.92–1.74)	0.148	0.78 (0.72–0.84)	349.0	0.129
MBP_max-min_	1.03 (0.75–1.40)	0.864	0.77 (0.71–0.84)	347.0	0.727
MBP_SD_	1.08 (0.80–1.47)	0.621	0.77 (0.71–0.84)	347.1	0.480
MBP_CV_	1.04 (0.76–1.41)	0.827	0.77 (0.71–0.84)	347.1	0.709
MBP_SV_	1.27 (0.94–1.73)	0.122	0.78 (0.71–0.84)	343.0	0.125

^*^p < 0.05.

BPV parameters were standardized (z-score) prior to regression analyses. Odds ratios (ORs) and 95% confidence intervals (CIs) were calculated per 1-standard deviation (SD) increase in each BPV parameter. A separate multivariable logistic regression model was constructed for each BPV parameter. The reported C-statistics were calculated from the predicted probabilities of the complete adjusted model, and the Akaike Information Criterion (AIC) and likelihood ratio test (LRT) were derived from the corresponding multivariable logistic regression model. All models were adjusted for variables with a *p* < 0.2 in the univariable analysis (intravenous thrombolysis, good collateral circulation, the Alberta Stroke Program Early CT Score [ASPECTS], National Institutes of Health Stroke Scale [NIHSS], moderate-to-severe cerebral small vessel disease, sustained major vessel patency, and atrial fibrillation).

BPV, blood pressure variability; PH, parenchymal hematoma; SBP, systolic blood pressure; DBP, diastolic blood pressure; PP, pulse pressure; MBP, mean blood pressure; max, maximum; min, minimum; max-min, the difference between maximum and minimum; SD, standard deviation; CV, coefficient of variation; SV, successive variation.

### 3.3 Interaction between PP_SV_ and persistent vessel patency or CSVD burden on PH

Interaction effects between PP_SV_ and persistent vessel patency or CSVD burden on PH were assessed by including interaction terms (PP_SV_ × persistent vessel patency and PP_SV_ × CSVD burden) in multivariable logistic regression models. Significant effect modification was observed for both persistent vessel patency (*P*interaction = 0.003) and CSVD burden (*P*interaction = 0.007). Complete adjusted interaction models are presented in Table 4. To further characterize these interaction effects, stratified analyses were performed according to persistent vessel patency (persistent major vessel occlusion vs. sustained major vessel patency) and CSVD burden (none-to-mild vs. moderate-to-severe).

**Table 4:** Complete multivariable interaction models evaluating the associations of PP_SV_ with parenchymal hematoma according to sustained major vessel patency and CSVD burden.

Variables	Interaction model 1 Interaction with sustained major vessel patency			Interaction model 2 Interaction with moderate-to-severe CSVD		
	*β*	Adjusted OR (95% CI)	*p* value	*β*	Adjusted OR (95% CI)	*p* value
Intravenous thrombolysis	0.487	1.627 (0.843–3.139)	0.147	0.598	1.819 (0.939–3.523)	0.076
Good collateral circulation	−0.185	0.831 (0.407–1.696)	0.611	−0.228	0.796 (0.394–1.609)	0.525
ASPECTS	−0.214	0.807 (0.707–0.923)	0.002	−0.189	0.827 (0.726–0.943)	0.004
NIHSS	−0.002	0.998 (0.940–1.059)	0.943	0.007	1.008 (0.949–1.069)	0.806
Moderate-to-severe CSVD	−1.169	0.311 (0.162–0.598)	<0.001	−1.528	0.217 (0.111–0.423)	<0.001
Sustained major vessel patency	−1.370	0.254 (0.114–0.564)	0.001	−1.655	0.191 (0.085–0.430)	<0.001
Atrial fibrillation	−0.811	0.445 (0.218–0.905)	0.025	−0.822	0.439 (0.219–0.883)	0.021
PP_SV_ (per 1-SD increase)	−0.200	0.819 (0.422–1.589)	0.555	0.964	2.621 (1.746–3.934)	<0.001
**Interaction term**	**1.156**	**3.178 (1.476–6.846)**	**0.003**	**−0.874**	**0.417 (0.222–0.784)**	**0.007**

PP_SV_, pulse pressure successive variation; OR, odds ratio; CI, confidence interval; ASPECTS, Alberta Stroke Program Early CT Score; NIHSS, National Institutes of Health Stroke Scale; CSVD, cerebral small vessel disease.

PP_SV_ was standardized (z-score) before analysis. Odds ratios are presented per 1-standard deviation increase in PPSV. Model 1 included an interaction term between PP_SV_ and sustained major vessel patency, whereas Model 2 included an interaction term between PP_SV_ and moderate-to-severe CSVD. Both models were adjusted for intravenous thrombolysis, good collateral circulation, ASPECTS, NIHSS, moderate-to-severe CSVD, sustained major vessel patency, and atrial fibrillation. Bold values indicate the estimates and p values for the interaction terms.

### 3.4 Stratified analyses

**Figure 1:**
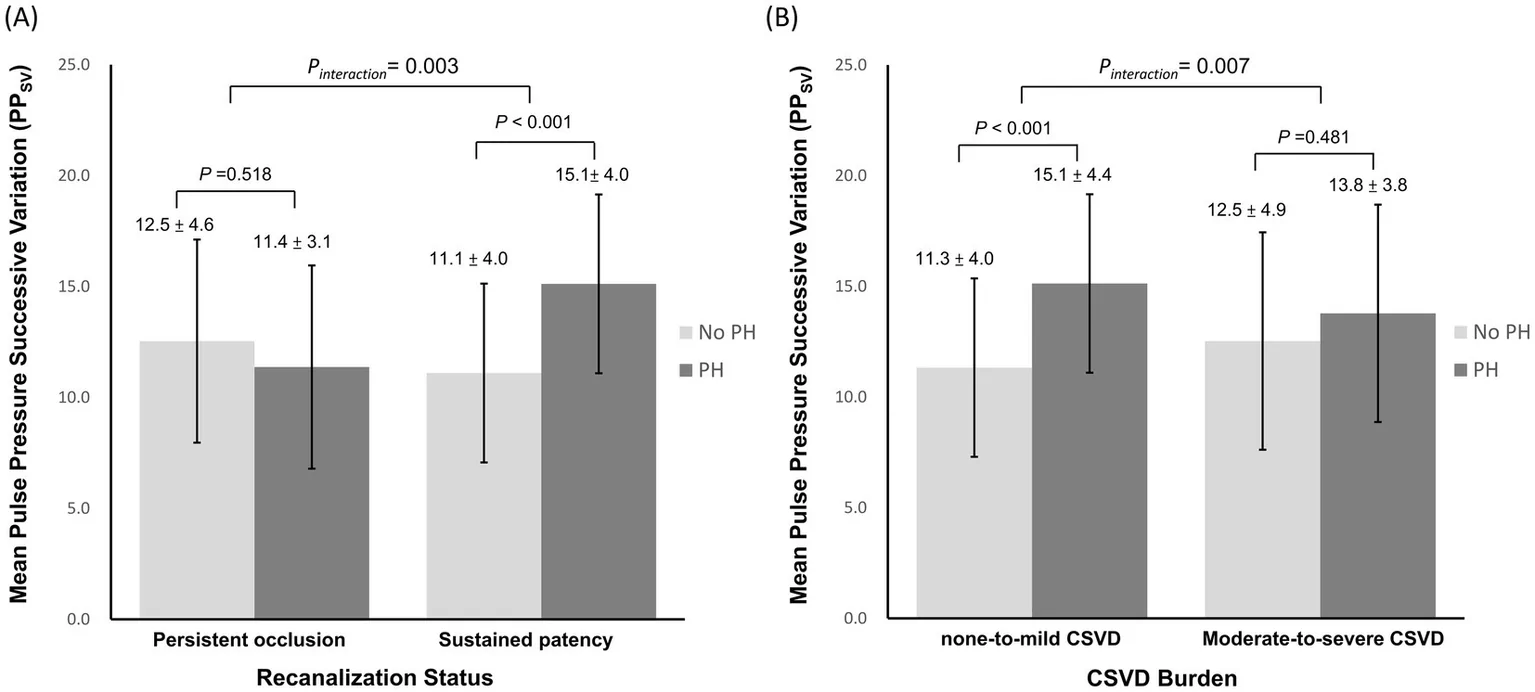
Association between mean pulse pressure successive variation (PP_SV_) and Parenchymal Hemorrhage (PH) stratified by persistent vessel patency and Cerebral Small Vessel Disease (CSVD) burden. **(A)** Comparison of mean PP_SV_ between patients with and without PH stratified by persistent vessel patency. A significant interaction was observed (*P*_interacti*on*_ = 0.003), where higher PP_SV_ was significantly associated with PH only in patients with sustained major vessel patency. No significant difference was observed in patients with persistent major vessel occlusion. **(B)** Comparison of mean PP_SV_ between patients with and without PH stratified by CSVD burden. A significant interaction was also observed (*P*_interacti*on*_ = 0.007). Compared to patients without PH, PP_SV_ was significantly higher in patients with PH in those with none-to-mild CSVD, whereas no significant difference was observed in those with moderate-to-severe CSVD. The data are presented as the mean ± SD. *p* values represent between-group comparisons within each stratum. *p* for interaction (*P*_interacti*on*_) indicates effect modification by persistent vessel patency and CSVD burden. PP_SV_, pulse pressure successive variation; PH, parenchymal hemorrhage; CSVD, cerebral small vessel disease; *P*_interacti*on*_, *p* for interaction.

In stratified analyses, patients with sustained major vessel patency who developed PH had a significantly higher mean PP_SV_ compared to those without PH (15.1 ± 4.0 vs. 11.1 ± 4.0; *p* < 0.001), and a higher PP_SV_ was independently associated with an increased risk of PH in this subgroup (OR, 2.67 [95% CI, 1.79–3.97]; *p* < 0.001). However, no significant relationship or elevation in PP_SV_ was observed among patients with persistent major vessel occlusion (OR, 0.80 [95% CI, 0.42–1.56]; *p* = 0.518). When stratified by CSVD burden, patients with none-to-mild CSVD who developed PH had substantially higher mean PP_SV_ compared to those without PH (15.1 ± 4.4 vs. 11.3 ± 4.0; *p* < 0.001), and a higher PP_SV_ was independently associated with an increased risk of PH (OR, 2.61; 95% CI, 1.74–3.94; *p* < 0.001). Conversely, no such association was observed in the moderate-to-severe CSVD subgroup (OR, 1.22 [95% CI, 0.71–2.10]; *p* = 0.481) (Figure 1; Supplementary Figure S1).

## 4 Discussion

This study showed that increased BPV within the first 24 h following EVT, particularly PP variability, was independently associated with PH. Among the BPV parameters, PP_SV_ demonstrated favorable overall model performance. Importantly, the association between BPV and PH was not uniform across all patients but was significantly modified by both persistent vessel patency and the CSVD burden. These findings suggest that individualized rather than universal blood pressure management strategies may be warranted during the critical 24-h period following thrombectomy.

Ischemic injury can damage endothelial cells and disrupt BBB integrity, allowing free movement of particles across the barrier and diminishing the osmotic gradient. Under these conditions, blood pressure, as the primary determinant of hydrostatic pressure, becomes the principal driving force of blood extravasation into infarcted tissue (2, 3). Increased BPV may impose repetitive pulsatile mechanical stress on fragile capillary beds, potentially aggravating microvascular injury and facilitating the progression from minor leakage to PH (4). These mechanisms provide a biological rationale for the independent association between post-thrombectomy BPV and PH.

PP reflects the pulsatile component of systemic circulation and is closely related to arterial stiffness (31). Increased PP may induce pathological stretching of cerebral vessels and contribute to endothelial dysfunction, ultimately promoting BBB disruption (32, 33). Previous studies have reported associations between elevated PP and intracranial hemorrhage in patients with ischemic stroke and in those with myocardial infarction receiving thrombolytic therapy (31, 33, 34). In addition, PP-derived BPV parameters are more consistently associated with functional outcomes and show better discrimination compared with SBP-derived parameters (35). In the present study, several PP-derived BPV parameters, including PP_max–min_, PP_SD_, PP_CV_, and PP_SV_, were independently associated with PH. Taken together, these findings support the concept that excessive arterial pulsatility may increase the risk of hemorrhagic complications after ischemic stroke.

Among the BPV metrics examined, SV-based parameters showed the most consistent relationship with PH. PP_SV_ was independently associated with PH, whereas SBP_SV_ and DBP_SV_ demonstrated a trend toward this association. In contrast, dispersion-based metrics such as SD and CV were not significantly associated with PH. Previous studies have shown that SV-derived BPV parameters are associated with hemorrhagic transformation and PH after ischemic stroke, regardless of whether thrombolytic therapy was administered (4, 8). Additionally, a meta-analysis suggested that SBP_SV_ may have a stronger effect on functional outcomes than SBP_SD_ or SBP_CV_ do (36). Another meta-analysis found that post-thrombectomy SBP variability, assessed by SBP_SD_ and SBP_CV_, was associated with mortality and functional disability but not with symptomatic intracranial hemorrhage (sICH) (37). In our study, however, the associations with PH were mainly observed for PP-derived BPV, particularly PP_SV_. These studies differed in several respects. SD and CV reflect overall BP dispersion, whereas SV reflects fluctuations between consecutive BP measurements. Moreover, PP reflects the pulsatile component of systemic circulation and may capture hemodynamic characteristics different from those reflected by SBP. Differences in BPV metrics, hemorrhagic outcomes (sICH vs. imaging-defined PH), and BP sampling protocols may therefore partly account for the different findings across studies. However, these differences do not establish the superiority of one BPV metric over another. Taken together, these observations suggest that SV, by reflecting fluctuations between consecutive BP measurements, may capture a distinct component of short-term hemodynamic instability that warrants further investigation in relation to hemorrhagic complications after EVT.

Our findings should be interpreted in light of current evidence on post-thrombectomy BP management. The updated ESO and AHA/ASA guidelines recommend against intensive SBP lowering below 140 mmHg during the early post-thrombectomy period (5, 6). The relatively low mean SBP in our cohort was observed under non-standardized BP management during the retrospective study period.

Importantly, our study evaluated BPV rather than a specific BP target or BP-lowering strategy; therefore, our findings should not be interpreted as supporting intensive BP lowering. Whether BPV represents a modifiable therapeutic target remains uncertain and requires prospective investigation.

In addition to ischemic injury itself, reperfusion following recanalization with persistent vessel patency may further exacerbate BBB disruption through oxidative stress and inflammatory cascades (2, 15). According to the results of the stratified analyses, higher PP_SV_ was associated with PH in patients with sustained major vessel patency, whereas no significant association was observed in those with persistent major vessel occlusion. These findings may be explained by the hemodynamic consequences of reperfusion. Restoration of antegrade blood flow may increase the transmission of systemic pulsatile stress to the injured microvasculature, rendering it more susceptible to blood extravasation. Conversely, when major vessel occlusion persists, the downstream microcirculation may remain relatively shielded from systemic pressure fluctuations, thereby attenuating the effect of BPV on hemorrhagic complications after ischemic stroke. In patients undergoing EVT, successful recanalization has been associated with an increased risk of symptomatic intracranial hemorrhage (38, 39). Moreover, our previous work demonstrated that BPV was associated with an increased risk of PH in patients with atrial fibrillation-related stroke, with this association observed only in those with recanalization (40). Taken together, these findings support a potential role of reperfusion in modulating this effect of BPV on hemorrhagic complications.

In the present study, although a moderate-to-severe CSVD burden was more prevalent in patients with PH, the association between increased PP_SV_ and PH was predominantly observed in patients with none-to-mild CSVD. This pattern may reflect a potential “ceiling effect” of BBB fragility in advanced microvascular disease. Prior studies have shown that a greater CSVD burden is associated with increased BBB permeability (16, 19). In patients with moderate-to-severe CSVD, chronic endothelial dysfunction may already lead to substantial baseline BBB impairment, thereby predisposing them to blood extravasation even in the absence of marked blood pressure fluctuations. In contrast, in patients with none-to-mild CSVD, the BBB is relatively preserved but may be more vulnerable to repetitive mechanical stress induced by increased BPV.

There were several limitations in this study. First, given its retrospective design, a causal relationship between BPV and PH cannot be definitively established. In addition, 90-day functional outcome data were not consistently available, precluding evaluation of the relationships among BPV, PH, and long-term functional outcomes. Second, blood pressure was measured at 2-h intervals rather than by continuous monitoring, which may have led to an underestimation of short-term blood pressure fluctuations occurring within shorter time frames. Third, there is potential concern regarding optimism bias and overfitting because the same dataset was used to select PP_SV_ and to perform the subsequent analyses. Fourth, atrial fibrillation was prevalent in this cohort and may have influenced oscillometric blood pressure measurements and PP_SV_ estimation. Although atrial fibrillation was included as a covariate in the multivariable models, its potential influence on oscillometric BP measurement and PP_SV_ estimation cannot be completely excluded. Finally, selection bias related to MRI availability cannot be excluded. Patients without adequate MRI were excluded because reliable assessment of CSVD burden was not possible, and a *post hoc* review suggested that MRI unavailability preferentially affected patients with more severe clinical conditions, potentially limiting the generalizability of our findings.

In conclusion, increased BPV within the first 24 h following EVT, particularly PP variability, was independently associated with PH. This association was most evident in patients with sustained major vessel patency and those with none-to-mild CSVD burden, suggesting a role for both macrovascular persistent vessel patency and underlying microvascular BBB integrity. These findings suggest that PP_SV_ may represent a potential marker for risk stratification after EVT. However, they should not be interpreted as supporting intensive BP lowering or a specific BP target. Further prospective studies are warranted to validate the clinical significance of BPV and to determine whether BPV represents a modifiable therapeutic target after EVT.

## Data Availability

The datasets generated and/or analyzed during the current study are not publicly available due to patient privacy and ethical considerations but are available from the corresponding author on reasonable request, subject to applicable institutional and ethical requirements.
