# Supplementary material for: Blood pressure variability and parenchymal hematoma after endovascular thrombectomy: effect modification by recanalization status and cerebral small vessel disease burden
Source: Front Neurol. 2026 Sep 10;17:1927081. doi: 10.3389/fneur.2026.1927081 (PMC13600807; doi:10.3389/fneur.2026.1927081)

Supplementary Material

Supplementary Figure S1.

Forest Plot of Interaction Effects between Pulse Pressure Successive Variation (PP_{SV}) and Parenchymal Hematoma (PH) Stratified by Recanalization Status and Cerebral Small Vessel Disease (CSVD) Burden

Multivariable logistic regression models demonstrated interaction effects between PP_{SV} and both recanalization status ($P_{interaction}$ = 0.003) and CSVD burden ($P_{interaction}$ = 0.007) on the risk of PH. In stratified analyses, higher PP_{SV} was associated with PH in patients with sustained major vessel patency (adjusted OR, 2.67 [95% CI, 1.79–3.97]; $P < 0.001$), whereas no significant association was observed in those with persistent major vessel occlusion. Additionally, higher PP_{SV} was associated with PH in patients with none-to-mild CSVD (adjusted OR, 2.61 [95% CI, 1.74–3.94]; $P < 0.001$), but not in those with moderate-to-severe CSVD.

Adjusted odds ratios (ORs) with 95% confidence intervals (CIs) were estimated per 1–standard deviation increase in PP_{SV}. All models were adjusted for intravenous thrombolysis, good collateral circulation, Alberta Stroke Program Early CT Score (ASPECTS), National Institutes of Health Stroke Scale (NIHSS), and atrial fibrillation. In analyses stratified by recanalization status, additional adjustment for CSVD burden was performed; conversely, analyses stratified by CSVD burden included additional adjustment for recanalization status. A two-sided $P < 0.05$ was considered statistically significant.

Abbreviations:

PP_{SV}, pulse pressure successive variation; PH, parenchymal hematoma; CSVD, cerebral small vessel disease

Subgroups	n	PH Events (%)	Adjusted OR (95% CI)	*P* value	$P_{interaction}$
**Recanalization status**					0.003
Persistent major vessel occlusion	98	15 (15.3%)	0.80 (0.42 – 1.56)	0.518	
Sustained major vessel patency	204	63 (30.9%)	2.67 (1.79 – 3.97)	< 0.001	
**CSVD burden**					0.007
None-to-mild CSVD	203	36 (17.7%)	2.61 (1.74 – 3.94)	< 0.001	
Moderate-to-severe CSVD	99	42 (42.4%)	1.22 (0.71 – 2.10)	0.481	

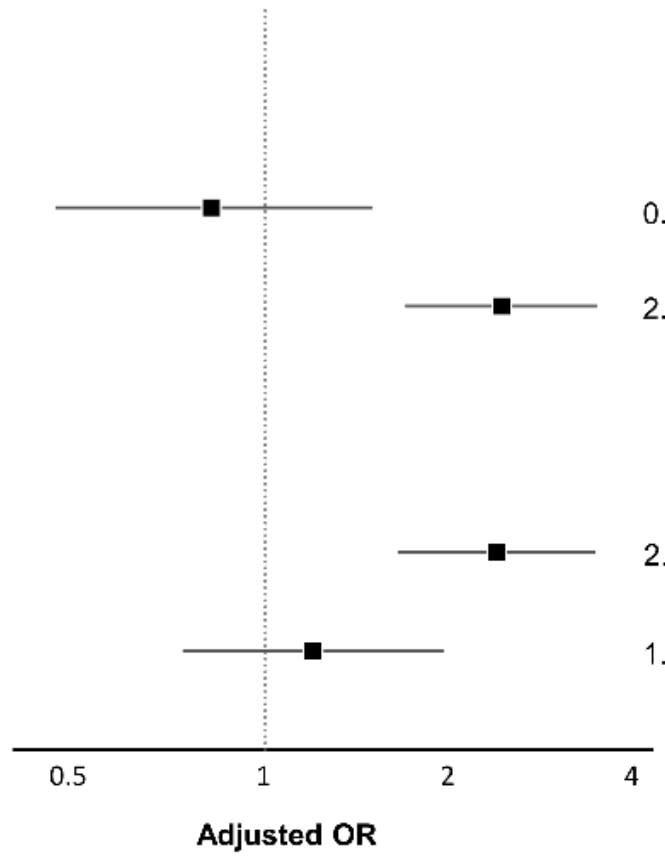

Supplement: Supplementary file 1 [file Image_1.pdf]
